# Modeling and Simulation of Lipid Membranes

**DOI:** 10.3390/membranes12060549

**Published:** 2022-05-25

**Authors:** Jordi Martí, Carles Calero

**Affiliations:** 1 Department of Physics, Polytechnic University of Catalonia-Barcelona Tech, B5-209 Northern Campus UPC, 08034 Barcelona, Spain; jordi.marti@upc.edu; 2 Department of Condensed Matter Physics, University of Barcelona, Carrer de Martí i Franquès, 1, 08028 Barcelona, Spain

Cell membranes separate the interior of cells and the exterior environment, providing protection, controlling the passage of substances, and governing the interaction with other biomolecules and signalling processes. They are complex structures that, mainly driven by the hydrophobic effect [1], are based upon phospholipid bilayer assemblies containing sterols, glycolipids, and a wide variety of proteins located both at the exterior surface and spanning the membrane [2,3]. There exist a large number of different types of phospholipids, each with a given function, although we understand only a small fraction of them [4]. Recently, studies of the physical and biochemical characteristics of lipid molecules as been referred to as lipidomics [5] in recognition of their fundamental importance for the understanding of cell biology.

Over the years, a great variety of experimental techniques have been developed to investigate the structure, dynamics and function of phospholipid membranes. These include nuclear magnetic resonance [6], X-ray scattering [7], small angle and quasi-elastic neutron scattering spectroscopy [8], scanning tunneling microscopy [9], and more recently new techniques to probe previously unaccessible length- and time-scales, such as stimulated emission depletion microscopy-fluorescence correlation spectroscopy [10], terahertz time-domain spectroscopy [11], or microfluidic techniques [12], to mention just a few. In parallel, in recent decades the increase of computer power and the development of new modeling and simulation techniques have allowed a significant improvement in the theoretical description of lipid membranes. As a consequence, plenty of papers have been devoted to the modeling and simulation of cell membranes, from pioneering works at the atomic level of description [13,14,15] to a multiplicity of coarse-grained approaches [16], the latter allowing to run for long simulations over larger and larger time and distance scales and to study processes such as lipid rafts [17] or full membrane dynamics [18]. Indeed, computer simulations provide relevant information on the structure and dynamics of lipid membranes, and can be used to complement and interpret the experimental data, which is limited by the length and time resolution of the experiment.

This Special Issue of *Membranes* discusses recent progress in the study of membrane systems mainly using computational (usually molecular dynamics) or mixed methodologies. It contains eight research articles. The complete description of each study and the main results are presented in more detail in the full manuscript, which the reader is invited to read. A brief summary of the articles is presented as follows.

Sessa et al. [19] investigate with a combination of permeability experiments and molecular dynamics simulations the crucial issue of the interaction between proteins and phospholipid membranes. The authors compare the effects on a model lipid bilayer of a natural peptide and an analog synthetic peptide which contains a highly hydrophobic azobenzene group. Their computer simulations suggest that the affinity of the peptide is significantly enhanced by the inclusion of such residue. In addition, simulations and experiments on the entrapment capacity of large vesicles show that the modified peptide induces a larger perturbance on the structure of the lipid bilayer, increasing its permeability. Understanding this effect may be important for the design of new peptides with specific functionalities with potential therapeutic applications.

The article by Lu and Marti [20] highlights the influence of cholesterol in the orientations and structural conformations of the oncogene KRas-4B. This protein is well known for its extended presence in a wide variety of cancers and because of its undruggability. The authors have performed microsecond molecular dynamics simulations using the CHARMM36 force field to observe that high cholesterol contents in the cell membrane favor a given orientation with the protein exposing its effector-binding loop for signal transduction and helping KRas-4B mutant species to remain in its active state. This suggests that high cholesterol intake will increase mortality of some cancer patients.

The next contribution was due to Aragon-Muriel et al. [21] and it reports a study of a newly designed Schiff base derivative from 2-(m-aminophenyl)benzimidazole and 2,4-dihydroxybenzaldehyde interacting with two synthetic membrane models prepared with pure 1,2-dimyristoyl-sn-glycero-3-phosphocholine and a 3:1 mixture of this lipid with 1,2-dimyristoyl-sn-glycero-3-phosphoglycerol, in order to mimic eukaryotic and prokaryotic membranes. The study was performed by means of a combined in vivo-in silico study using differential scanning calorimetry, spectroscopic and spectrometric techniques and molecular dynamics simulations. The main results indicate that the Schiff derivative induces higher fluidity at the mixed membrane. As a second part of their study, the authors modeled an erythrocyte membrane model formed by 1-palmitoyl-2-oleoyl-sn-glycero-3-phosphoethanolamine, N-(15Z-tetracosenoyl)-sphing-4-enine-1-phosphocholine and 1-palmitoyl-2-oleoyl-sn-glycero-3-phosphocholine and observed that the Schiff derivative showed high affinity to the different membranes due to hydrophobic interactions or hydrogen bonds.

The interplay between scattering experiments and molecular dynamics simulations to obtain information on the structure of model phospholipid membranes is discussed in the article [22]. Zec and co-workers provide a detailed comparison between the results of scattering experiments (neutron and X-ray reflectometry and small angle scattering measurements) and calculated values obtained from standard all-atom MD simulations of bilayers composed of popular phospholipids (1,2-dimyristoyl-sn-glycero-3-phosphocholine (DMPC) and 1,2-dilinoleoyl-sn-glycero-3-phosphocholine. The authors show that MD simulations can be used to interpret from a nanoscopic perspective the results from scattering experiments, which prove larger length and time scales. Their analysis also identifies the uncertainties and sources of error from scattering experiments and simulations, which need to be considered in order to draw significant conclusions from their comparison.

In the paper by Radhakrishnan et al. [23] the authors used molecular dynamics techniques in order to study the permeation of membranes by several relevant solutes, such as Withaferin A, Withanone, Caffeic Acid Phenethyl Ester and Artepillin C when they are at the interface of a cell membrane model formed by phosphatidylserine lipids. Their results indicated that exposure of phosphatidylserine can favor the permeation of Withaferin A, Withanone and of Caffeic Acid Phenethyl Ester through a cancer cell membrane when compared to a normal membrane. The authors showed the ability of phosphatidylserine exposure-based models for analyzing how cancer cells are able to perform drug selectivity.

In Reference [24], Trejo and co-workers review the main properties of red blood cells’ (RBC) membranes and their effect on blood rheology. The authors describe the mechanical properties of RBC membranes and the mesoscopic theory to model their relevant elastic features, as well as the resulting membrane dynamics. They also discuss the interaction of RBCs with the constituents of blood plasma through the membrane, of great importance to understand RBCs mutual interactions and the formation of RBCs aggregates. The consequences of RBCs properties on fluid dynamics of blood in the circulatory system (hemodynamics) are also reviewed, giving an account of recent advancements in numerical and experimental techniques which have provided new information on the subject. In particular, Trejo et al. review in detail the use of recent microfluidic techniques to obtain information on the properties of single RBCs as well as on collective effects which determine the rheological properties of blood (hemorheology). Finally, a review of the disorders which alter the hemodynamics and rheological properties of blood is provided, and an account is given of the microfluidic techniques developed for their diagnostic.

In the work of Hu and Marti [25], the authors reported a molecular dynamics study on the atomic interactions of a lipid bilayer membrane formed by dioleoylphosphatidylcholine, 1,2-dioleoyl-sn-glycero-3-phosphoserine and cholesterol with a series of derivatives of the drug benzothiadiazine, designed in silico, all within a potassium chloride aqueous solution. The benzothiadiazine derivatives were obtained by single-hydrogen site substitution and it has been revealed that all them have strong affinity to remain at the cell membrane interface, with variable residence times in the range 10–70 ns. The authors observed that benzothiadiazine derivatives can bind lipids and cholesterol chains with single and double hydrogen-bonds of rather short characteristic lengths.

The influence of the membrane on the properties of transmembrane proteins is investigated by Asare and co-workers using numerical simulations [26]. The authors perform MD simulations of KCNE3, a transmembrane protein associated with several potassium channels, inserted in different phospholipid bilayers: DMPC, 1-palmitoyl-2-oleoyl-sn-glycero-3-phosphocholine (POPC), and a mixture of POPC and POPG (1-Palmitoyl-2-oleoyl-sn-glycero-3-phosphatidylglycerol) in a 3:1 proportion, to study how such environments determine its structural and dynamical properties. Their simulations indicate that the central part of the protein immersed in the membrane, the transmembrane domain, is more rigid and stable than the two ends of the protein which are surrounded by the electrolyte. The results reported by Asare and co-workers can help complement the information extracted from experiment on KCNE3’s function in its native membrane environment.

Despite studies of model lipid membranes have been carried out for long time, there are still many aspects and theoretical findings that have not been yet verified experimentally and for which the existing results are incomplete or inconsistent. Conversely, there are also experimental results which still lack of appropriate microscopical interpretation. Therefore, the main objective of this Special Issue was to collect a sample of recent scientific works on the modeling and simulation lipid membranes, with special aim in the interactions of the two principal techniques (theory-simulation vs. experiments) and their mutual benefit. The techniques presented here, from purely computational to the mixture of simulation and experimental methods in some cases, have helped us to understand essential physical properties as the structure and dynamics of specific lipid membranes and solutes. These studies will provide new insights into the fundamental principles underlying physiological functions of cell membranes and their relationship with other components of cells and tissues. We believe that this objective has been successfully achieved, for which we express our heartfelt appreciation to all authors and reviewers for their excellent contributions. 

## Data Availability

Not applicable.
